# Risk assessment of high concentrations of molybdenum in forage

**DOI:** 10.1007/s10653-018-0132-x

**Published:** 2018-06-19

**Authors:** U. Axelson, M. Söderström, A. Jonsson

**Affiliations:** 1 Precision Agriculture and Pedometrics, Department of Soil and Environment, Swedish University of Agricultural Sciences, PO Box 234, 523 23 Skara, Sweden; 2 The Rural Economy and Agricultural Society, Järnvägsgatan 18, 532 30 Skara, Sweden; 3 RISE Agrifood and Biocience, PO Box 63, 523 21 Skara, Sweden

**Keywords:** Molybdenum, Ruminants, Forage, Risk assessment

## Abstract

Molybdenum is toxic to ruminants when present in high levels in forage, causing physiological copper deficiency. A critical level for ruminants is 3–10 mg Mo kg^−1^ dry matter. The average Mo level varies considerably between different arable soils, depending mainly on soil parent material. This study investigated the possibility of using various existing sources of geospatial information (geophysical, biogeochemical and soil chemical) to develop a geography-based risk assessment system. Forage samples (*n* = 173) were collected in 2006–2007. Three types of national geoscientific datasets were tested: (1) *SEPA topsoil*, comprising data from arable land within the Swedish environmental monitoring programme; (2) *SGU biogeochemical*, containing data from aquatic plant root material collected in small streams; and (3) *SGU geophysical*, consisting of data from airborne gamma-ray scanning. The digital postcode area map was used for geocoding, with Mo concentrations in forage assigned to arable parts of the corresponding postcode area. By combining this with the three national geoscientific databases, it was possible to construct a risk map using fuzzy classification depicting *High-risk, Intermediate-risk, Low-risk* and *Very-low-risk* areas. The map was validated using 42 randomly selected samples. All samples but one with Mo > 3 mg kg^−1^ were found in postcode areas designated *High risk*. Thus, the risk map developed seems to be useful as a decision support system on where standard forage analyses need to be supplemented with Mo analyses.

## Introduction

Molybdenum (Mo) is toxic to ruminants when present in high concentrations in forage. Molybdenum itself is not toxic, but together with sulphur (S) it forms thiomolybdate, which can cause physiological copper (Cu) deficiency in ruminants (Gould and Kendall 2011; Merl et al. 2006). The reported critical dietary level of Mo in forage varies. A concentration of 5 mg Mo kg^−1^ dry matter (DM) is reported to be the upper limit by Gardner et al. (2003) and Majak et al. (2004), while Blood et al. (1979) report the interval 3–10 mg Mo kg^−1^ DM to be dangerous for ruminants. However, it is not the content of Mo alone that determines the effect on animals, but rather the mass ratio between Cu and Mo concentrations in forage. If the Cu/Mo ratio is < 2, there is a risk of what is called secondary Cu deficiency or molybdenosis. Gupta and Gupta (1998) set the limit for the Cu/Mo ratio between 2 and 3, while Ward (1978) and Blood et al. (1979) consider a ratio of 2 to be critical.

According to Ward (1978), high Mo levels have two origins, namely high concentrations in soil due to parent materials and contamination from pollution, for example, by mining wastes (Neunhäuserer et al. 2001). The average Mo levels in different arable soils vary considerably. The normal concentration of Mo in arable soils globally is about 1–2 mg kg^−1^ (Jones et al. 1990; Reddy et al. 1997). However, values as high as 100 mg kg^−1^ have been found in soils developed on black shales (Jones et al. 1990). Eriksson et al. (2010) reported an average concentration for 1535 topsoil samples in Sweden of 1.5 mg Mo kg^−1^, with a range of 0.1–81.8 mg kg^−1^. The highest concentrations were observed in areas with Cambro-Silurian sedimentary bedrock, specifically alum shales. Eriksson et al. (2017) detected concentrations of 0.2–10 mg kg^−1^ in Swedish soils, with higher levels in topsoil than in subsoil.

Solubility and uptake of Mo are dependent on chemical characteristics and processes in the soil (Alloway 1990; Reddy et al. 1997; Eriksson et al. 2017). Plants mainly absorb Mo as molybdate (MoO_4_^2−^) from the soil solution, and the molybdate form dominates in soil at pH values > 5 (Gupta 1997; Eriksson et al. 2017).

In a pilot study carried out in an area in south-west Sweden with high Mo levels, Mo concentrations > 5 mg kg^−1^ dry matter (DM) were found in 38 out of 95 forage samples from local farms (Axelson 2001, 2011). The highest observed content in forage was 58.7 mg Mo kg^−1^ DM, and the animals on that farm suffered from molybdenosis (Axelson 2011). Molybdenosis symptoms in wildlife (e.g. moose, *Alces alces* L.) have also been reported in this geographical region (Frank 1998).

In order to avoid the problem of excessive intake of Mo resulting in Cu deficiency in ruminants, it is important to develop tools that can be used by advisory services and farmers to facilitate identification of areas with a risk of high levels of Mo in animal feed. To achieve this, a better understanding is needed of correlations between Mo concentrations in soil and uptake in forage crops. The objective of the present study was thus to investigate the possibility of using various existing sources of geospatial (geophysical, biogeochemical and soil chemical) data to develop a geography-based risk assessment system for Mo in forage in Sweden that could serve as a decision support system (DSS) for advisors and farmers.

## Materials and methods

### Molybdenum content in forage

Data on Mo concentrations in forage samples from Swedish farms were provided by the analytical laboratory, Eurofins Agro Testing AB (Kristianstad, Sweden). The samples had been collected in 2006–2007 in southern Sweden, the region in which most of the arable land in Sweden is located (about 95% or 2.4 million ha, here designated ‘the study area’). The samples were taken by farmers or advisers according to the instruction given by the analytical laboratory (http://grovfoder.eurofins.se/media/26022/provtagningsanvisning-grovfoder_ny.pdf). At the laboratory, the sample, approximately 1 kg FM, was dried, milled and sieved (0.8 mm). The data were chosen in a screening process whereby all samples with Mo content > 3 mg kg^−1^ DM were selected, whereas samples with < 3 mg Mo kg^−1^ DM were selected randomly among the other analyses available. In total, analytical data on 173 samples were used for initial comparisons of forage Mo concentrations in relation to data from geochemical and geophysical datasets. In addition, data for 42 randomly selected forage samples analysed by the same laboratory during 2012–2014 were obtained, for use as an independent validation dataset.

For determination of Mo and Cu in forage in the analytical laboratory, one gram of the sample is diluted in capsules in a microwave oven in nitric acid (HNO_3_) and hydrogen peroxide (H_2_O_2_). Final determination is made using an ELAN DRC-e ICP-MS (PerkinElmer, Waltham, Mass., USA) (Roland Svanberg, Eurofins, pers. comm. 2013).

### Geoscientific datasets used

Three types of national geoscientific datasets were available for this work: (1) *SEPA topsoil*, containing data from analyses of arable soil within the Swedish environmental monitoring programme for cropland run by the Swedish Environmental Protection Agency (SEPA, Stockholm, Sweden); (2) *SGU biogeochemical*, containing data from analyses of aquatic plant roots collected in small streams performed by the Geological Survey of Sweden (SGU, Uppsala, Sweden); and (3) *SGU geophysical*, consisting of data from airborne gamma-ray scanning (SGU, Uppsala, Sweden).

1. In the SEPA topsoil monitoring programme for arable land, a total of 2034 topsoil samples have been analysed, aiming to cover all arable land in Sweden (Eriksson et al. 2010). The sample network is in principle uniformly distributed (about 1 sample per 1300 ha), and samples were collected during the period 2001–2007. At all sampling points, nine subsamples from 0 to 20 cm depth were collected within a 3 -m radius circle and bulked into one sample for analysis. These soil samples have been analysed for a wide range of elements (Eriksson et al. 2010), but in this study only data on Mo concentrations were used. These were determined by ALS Scandinavia AB (Luleå, Sweden) according to Swedish standard method SS 02 83 11 (Eriksson et al. 2010).
2. The SGU biogeochemical dataset comprises data from analyses of aquatic plants (roots, bryophytes) in small, mainly first- and second-order, streams. Most samples are composed of roots of sedge (*Carex* L.), willow moss (*Fontinalis antipyretica* L.) and meadowsweet (*Filipendula ulmaria* L.) (Lax 2009). The samples are usually collected within a distance of 50–100 m within first- and second-order streams, in flowing water, and the data are intended to reflect upstream conditions in small drainage basins (Lax and Selinus 2005). Approximately 20% of samples are collected from higher-order streams (Lax 2009). Molybdenum is one of more than 30 elements analysed in the aquatic plants.
3. Data from SGU geophysical aerial gamma-ray spectrometry measurements encompass recorded concentrations of the natural occurring radioactive isotopes potassium (^40^K; expressed in %), uranium (^238^U; ppm) and thorium (^232^Th; ppm). Data collection has been going on since the 1960s, at a flight height of 30 or 60 m and with a line spacing of 200–800 m (Söderström and Eriksson 2013). Each recording is separated by about 17 m along the flight line, but with a large response area on the ground, in principle four times the flight height (IAEA 2003). Earlier studies have shown that this type of data can be used for locating soils affected by alum shale (Söderström and Eriksson 2013), which are reported to have some of the highest soil Mo concentrations in Sweden (Eriksson et al. 2017).

### Mapping and statistics

**Fig. 1:**
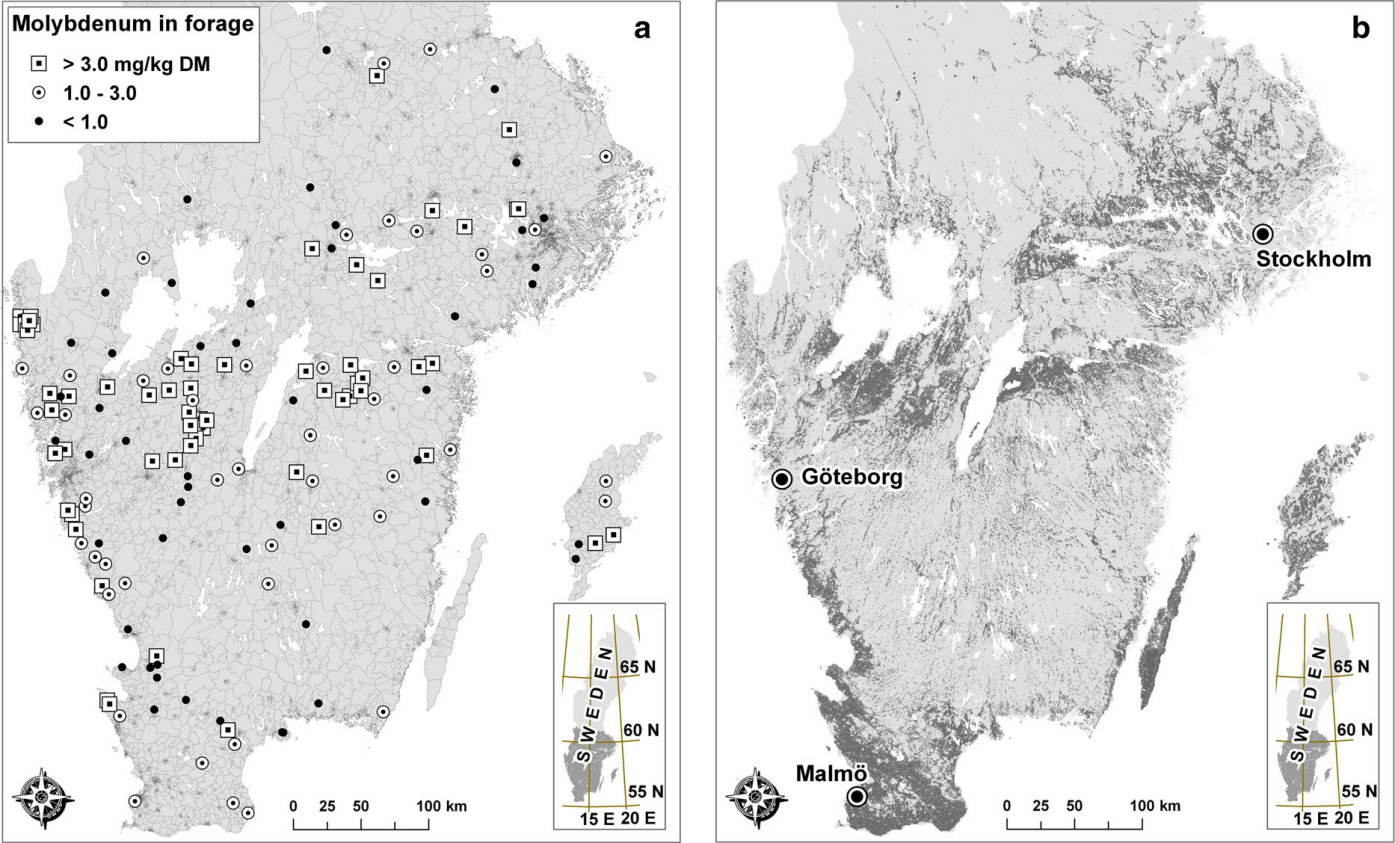
**a** Molybdenum analyses in forage. Postal code areas are shown in the background. **b** Arable land is shown in dark grey

The available information on geographical location of the Mo forage samples was their postcode. We used the digital postcode area map provided by Statistics Sweden (SCB, Örebro, Sweden) to geocode the forage samples (Fig. 1a). To remove all areas except arable land, the postcode area map was intersected with a detailed map of arable land from the EU subsidies database (Fig. 1b) provided by the Swedish Board of Agriculture (Jordbruksverket, Jönköping, Sweden). Postcode areas with at least 10 ha of arable land were retained for further analysis. (There were 3736 such postcode areas, with a median cropland acreage of 200 ha.) The data on Mo concentrations in forage provided by the laboratory were assigned to the polygon of arable land in the corresponding postcode area and classified into three risk classes: < 1 mg kg^−1^ (group 1); 1–3 mg kg^−1^ (group 2); and > 3 mg kg^−1^ (group 3). In ten cases, the postal address of forage analyses referred to postcode areas without arable land, and these samples were omitted from further analyses. One reason for this discrepancy could be that the owner of the field from which the forage sample was taken lived in a different postcode area. This could of course have been the situation in other cases, but it was judged to be relatively uncommon and of minor importance.

The three geoscientific datasets were interpolated using ordinary kriging with local variograms with the software Vesper (Whelan et al. 2001). The closest 100 observations to each postcode area were used for automatic estimation of sample variograms, fitting spherical variogram models to the data automatically by a nonlinear least squares approach, and predictions of the most likely value for each postcode area through ordinary kriging. Kriging is essentially a weighted moving average technique for estimation whereby weights are selected so that the estimation variance is minimised (Burrough and McDonnell 1998).

The maps constructed were used, individually and in combination, for comparisons with observed Mo content in forage. In order to combine the maps, we took the natural logarithm of the mapped soil variables to transform the positively skewed distributions to more normal distributions and used the continuous classification method fuzzy k-means (FKM; Burrough and McDonnell 1998), using the FuzME software (Minasny and McBratney 2002). This results in a set of *k* continuous classes (or clusters) in attribute space that minimises the within-class sum square error (Burrough and McDonnell 1998). In this method, every location can belong to any of *k* classes to some extent, with a value of likelihood, called a membership value (MF), ranging from 0 (no membership) to 1 (maximum membership), and the degree of fuzziness is set by the fuzzy exponent (*q*). To determine the optimal number of classes, the model with the lowest fuzzy performance index (Burrough and McDonnell 1998) was selected.

The Kruskal–Wallis test (Statistica 10; StatSoft Inc., Tulsa, USA) was used for evaluation of comparisons between Mo concentration classes in forage samples and the geoscientific datasets, individually and clustered with FKM. The Kruskal–Wallis test is the nonparametric analogue of a one-way ANOVA, but performed on ranked data (Kruskal and Wallis 1952), and is preferred when data are not normally distributed (Walpole et al. 2011). The Kruskal–Wallis test statistic H is computed according to:

1 $$ H = \frac{12}{{n\left( {n + 1} \right)}}\mathop \sum \limits_{i = 1}^{k} \frac{{R_{i}^{2} }}{{n_{i} }} - 3\left( {n + 1} \right), $$

where *n*_*i*_ (*i* = 1, 2,.., *k*) is the number of observations in each of the *k* groups and *R*_*i*_ is the sum of ranks for group *i*. This statistic approximates a *χ*^2^-distribution with *k*-1 degrees of freedom (d.f.). If H is greater than the *χ*^2^-per cent point function (α; d.f.), the null hypothesis that there is no significant difference between the groups is rejected.

## Results

A digital map of postcode areas (Statistics Sweden, Örebro, Sweden) in southern Sweden and the Mo content in forage observations are shown in Fig. 1a. There are 8740 postcode areas in this part of Sweden, but many are quite small and located within urban areas, and do not include any arable land. The extent of arable land is shown in Fig. 1b.

**Fig. 2:**
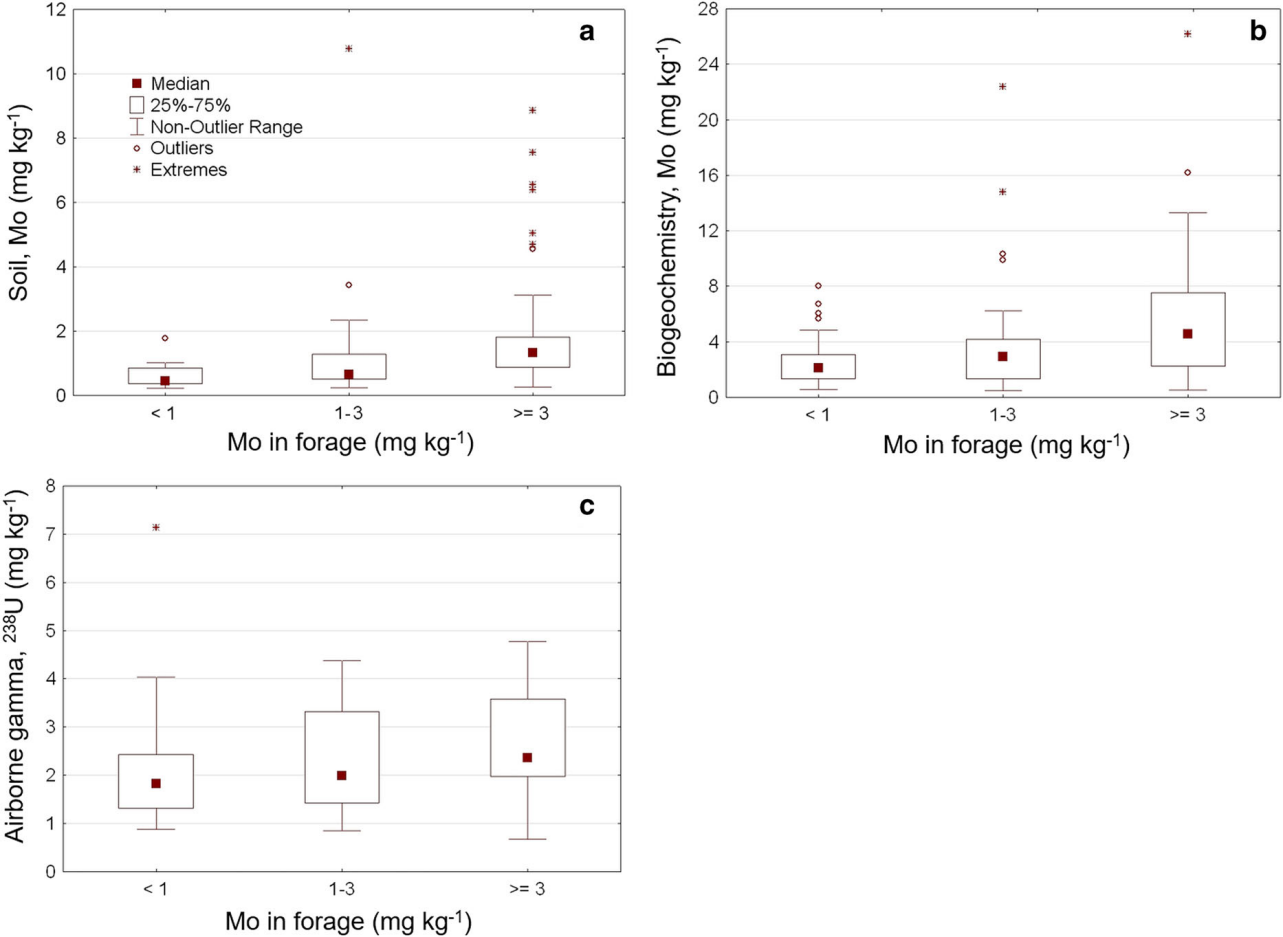
Relations between Mo in forage samples classified in three groups and three national geoscientific datasets displayed as boxplots:** a** SEPA topsoil, Mo in topsoil of arable land (Swedish Environmental Protection Agency 2010);** b** SGU biogeochemical, Mo in aquatic plants, (Geological Survey of Sweden);** c** SGU geophysical, 238U from the gamma-ray spectrometry database (Geological Survey of Sweden)

The Mo content in the forage samples was classified into three risk groups (groups 1–3 in Table 1). The relationships between Mo concentration in forage and Mo content in the soil from the three national databases, after classification into the three groups, are displayed in boxplots in Fig. 2a–c.

**Table 1:** Relation between Mo concentration in forage and the three geoscientific datasets

Mo in forage (mg kg^−1^)	SEPA topsoil arable land		SGU biogeochemical aquatic plants		SGU geophysical gamma ray	
	*n*	Mean rank	*n*	Mean rank	*n*	Mean rank
Group 1 (< 1)	46	48.3	46	57.4	46	64.7
Group 2 (1–3)	43	75.3	43	71.8	43	73.4
Group 3 (≥ 3)	74	106.8	74	103.2	74	97.8
Total n	163		163		163	
H (2 d.f.)		44.8		29.5		15.9
*p*		< 0.001		< 0.001		< 0.001

Summary of Kruskal–Wallis ANOVA by ranks and Mo content in forage samples classified in three groups

### Combining datasets for risk mapping

The risk classification from the fuzzy classification is given in Table 2. The values in Table 2 were qualitatively interpreted and classified according to the following criteria:

**Table 2:** Fuzzy classification at the postal code level clusters centre (left part) and a summary of corresponding molybdenum observations in forage (right part) for validation

Class	Clusters centre [ln (mg kg^−1^)]			Mo in forage (mg kg^−1^)				
	SEPA topsoil	SGU biogeochemical	SGU geophysical	*n*	Median	p25*	p75*	p90*
a	1.46	1.84	1.10	9	5.7	3.7	6.3	8.4
b	− 0.98	1.57	0.45	5	1.4	1.4	2.8	4.5
c	− 1.00	0.22	0.03	23	1.1	0.4	1.6	3.3
d	− 0.16	0.80	1.08	22	1.3	0.7	6.0	6.6
e	− 1.01	0.47	0.64	24	1.0	0.6	1.6	5.4
f	− 0.60	0.91	0.53	22	1.7	1.0	5.6	7.6
g	0.60	1.74	1.17	24	5.3	1.5	5.8	9.0

*p*m* refers to the *m*th percentile

- Class a, g = *High risk* (high median).
- Class d, f = *Intermediate risk* [low median; high 75th percentile (p*75*)]
- Class b, e = *Low risk* [low median; low 75th percentile; relatively high 90th percentile (p*90*)].
- Class c = *Very low risk* (low in general; Mo values > 3 mg kg^−1^ DM may occur).

**Fig. 3:**
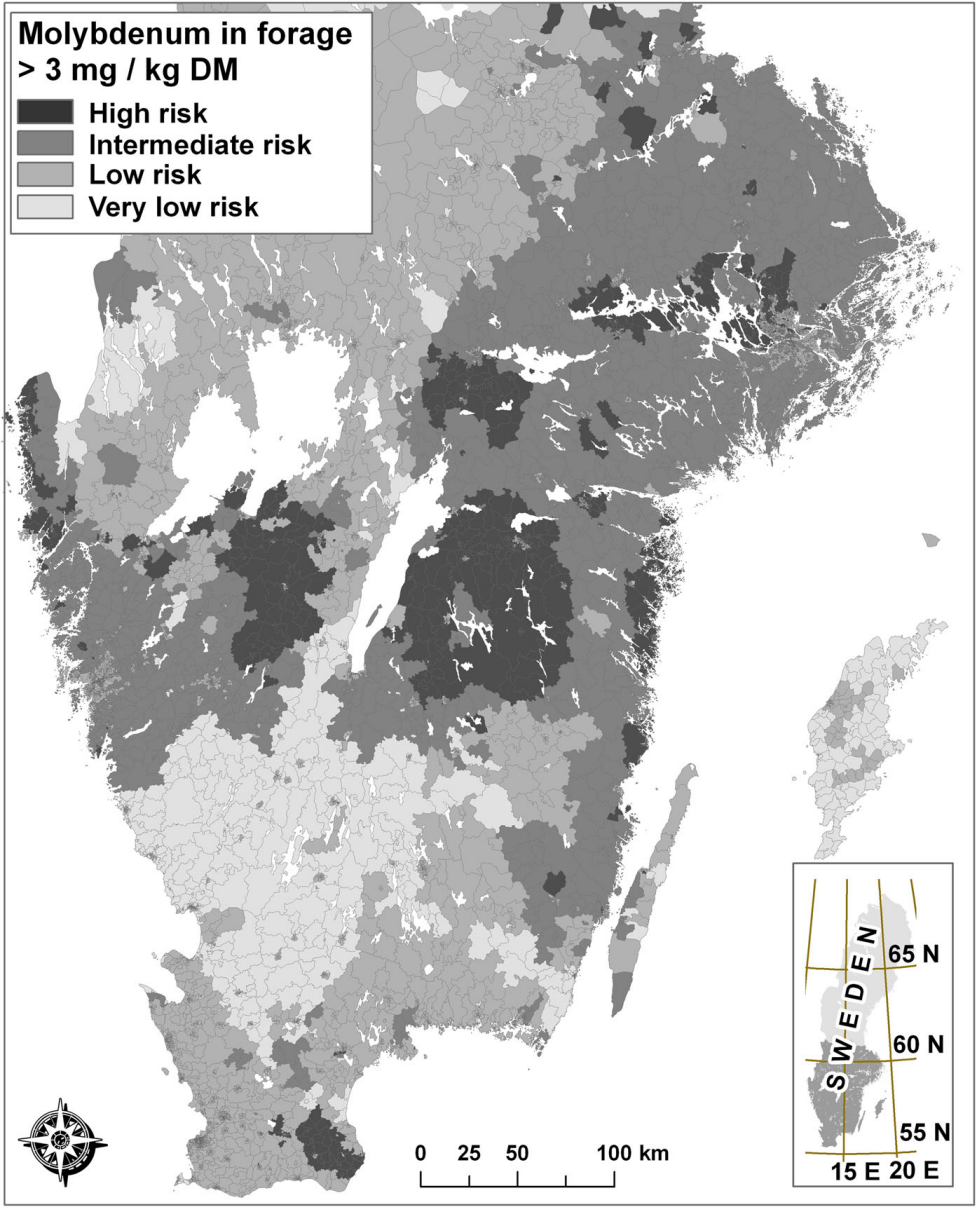
Resulting risk map for elevated Mo content in forage

All postcode areas were classified correspondingly, and a final general risk map was produced (Fig. 3). As is evident from Table 2, using a combination of all three geoscientific soil databases improved classification of the forage samples. The cluster centre of class (a) was characterised by high Mo concentrations in the SEPA topsoil database on arable land, high Mo content in aquatic plants in the SGU Biogeochemical database and high U content in the SGU Geophysical database. The Mo content in forage in class (a) areas was always relatively high. (Minimum value recorded was 2.03 mg kg^−1^ DM). Class (g), the other *High-risk* class, had somewhat lower values of Mo in the SEPA topsoil database, but the values in the other two databases were about the same as in class (a). The median, p*75* and p*90* of Mo concentration in forage were as high in class (g) as in class (a), but there were also a number of low Mo concentrations in forage (p*25* = 1.5). The lowest Mo content in forage was found in class (c) and was low in all three input databases. This was the only fuzzy class that was classified as *Very low risk*. Classes (b) and (e) also had a low Mo content in general, but at p*90* both classes had Mo values at or above 5 mg kg^−1^ DM. However, class (b) contained very few forage observations, so the validation statistics have a high degree of uncertainty for that class. Most of southern Sweden was classified as *Intermediate risk* (Fig. 3), corresponding to fuzzy classes (d) and (f). In those classes, the median Mo concentration in forage was low (1.3 and 1.7 mg kg^−1^ DM, respectively), but at p*75* of the observations the Mo concentration in forage was similar to that in areas classified as *High risk* (around 6 mg kg^−1^ DM).

**Fig. 4:**
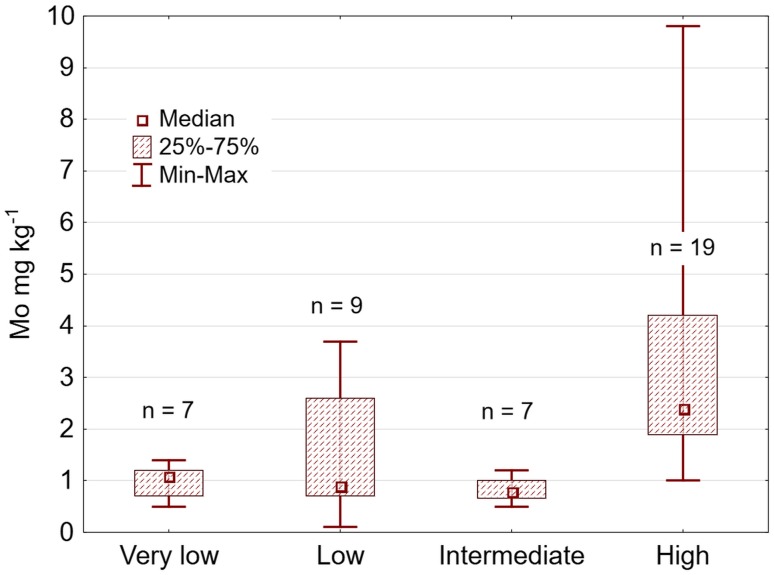
Validation of the risk map (Fig. 3) by 42 randomly selected Mo analyses

The risk map in Fig. 3 was validated against the 42 randomly selected samples analysed by Eurofins during 2012–2014. Only one sample allocated to the *Intermediate-risk*, *Low*-*risk* and *Very-low-risk* classes had a Mo content above > 3 mg kg^−1^. All the other samples with Mo > 3 mg kg^−1^ were from postcode areas designated as *High-risk* areas by the fuzzy risk model (Fig. 4). However, the differences between observed Mo values for the samples allocated to these three lower-risk classes did not entirely follow the order of classification. The class denominated *Low risk* had slightly higher forage Mo concentrations than the classes *Very low risk* and *Intermediate risk* and contained a few Mo values > 2 mg kg^−1^. However, the median of these classes was at an equally low level, around 1 mg Mo kg^−1^ DM, whereas the median of the *High-risk* class was 2.4 mg Mo kg^−1^ DM (and the mean was 3.1 mg Mo kg^−1^ DM).

## Discussion

Copper deficiency among ruminants is difficult to detect and often gives diffuse symptoms, and therefore, it is highly important for farmers to have knowledge of the risk situation prevailing on their particular farm. In order to obtain a complete picture of the risk scenario and conditions on each specific farm, it would be necessary to analyse the Mo concentration in soil from each field, since there is often great variation in Mo content within fields and between fields, as an effect of variation in soil parent material.

The hypothesis tested in this study was that the correlation between mapped Mo content in soil material and Mo content in plants can be used to predict a general regional risk of high uptake and high concentrations of Mo in forage. Such a risk map was successfully developed, using available geoscientific soil datasets, and used to identify areas with a high risk of elevated Mo concentrations in forage. In fact, with one single exception, validation samples that exceeded the critical level of > 3 mg Mo kg^−1^ DM were accurately assigned to areas designated *High risk* (Fig. 4).

All three national geoscientific databases tested gave similar trends. Statistical analysis and boxplots revealed a relationship between Mo concentration in forage and Mo content recorded in the databases. There were some differences in results between geoscientific datasets, which might be explained by the method of sample collection used for the various mappings. For example, samples in the SGU biogeochemical database are taken from aquatic plants growing in streams, which gives a correlation to Mo dissolved in soil drainage water. The strongest correlation with a single national database was achieved with the Swedish Environmental Protection Agency map (SEPA topsoil). This was not unexpected, since these samples were from the topsoil of arable land, whereas the other dataset more generally indicates the occurrence of Mo in the environment in the vicinity of arable land.

The validation results for a combination of the three databases using fuzzy classification were very encouraging. When the classification of postcode areas was compared against data from actual analyses, only one of the 42 samples in the validation dataset could be considered a false negative, i.e. a sample with Mo > 3 mg kg^−1^ allocated to an area classified as *Intermediate risk*. A similar method making use of this type of easy accessible data has been used previously in establishment of risk zones for high concentrations of cadmium (Cd) in crops and soil (Söderström and Eriksson 1996).

To get a complete picture of the risk scenario on a specific farm, it is advisable (or even essential) to make a complementary soil analysis of Mo in each field on the farm. This is especially important in areas with high variations in Mo concentration in the parent soil material. Since there is a connection between parent material and type of soil, a local bedrock map might also give some guidance on risk areas for high Mo levels in plants. As in the case of risk mapping of Cd (Söderström and Eriksson 2013), detailed maps at field level revealing the influence of fragments of alum shale can be produced with, e.g. vehicle-mounted gamma-ray sensors. That method should also be applicable for mapping of Mo.

In conclusion, we propose that risk maps such as that developed in this study can be used to give general recommendations on areas where standard forage analyses need to be supplemented with Mo analyses and other more detailed analyses on farms in High-risk areas.
